# Simultaneous Immobilization of Heavy Metals in MKPC-Based Mortar—Experimental Assessment

**DOI:** 10.3390/ma16247525

**Published:** 2023-12-06

**Authors:** Zbyšek Pavlík, Martina Záleská, Milena Pavlíková, Adam Pivák, Jana Nábělková, Ondřej Jankovský, Adéla Jiříčková, Oskar Chmel, Filip Průša

**Affiliations:** 1 Department of Materials Engineering and Chemistry, Faculty of Civil Engineering, Czech Technical University in Prague, Thákurova 7, 166 29 Prague, Czech Republic; martina.zaleska@fsv.cvut.cz (M.Z.); milena.pavlikova@fsv.cvut.cz (M.P.); adam.pivak@fsv.cvut.cz (A.P.); 2 Department of Sanitary and Ecological Engineering, Faculty of Civil Engineering, Czech Technical University in Prague, Thákurova 7, 166 29 Prague, Czech Republic; jana.nabelkova@fsv.cvut.cz; 3 Department of Inorganic Chemistry, Faculty of Chemical Technology, University of Chemistry and Technology, Technická 5, 166 28 Prague, Czech Republic; ondrej.jankovsky@vscht.cz (O.J.); adela.jirickova@vscht.cz (A.J.); oskar.chmel@vscht.cz (O.C.); filip.prusa@vscht.cz (F.P.); 4 Department of Metals and Corrosion Engineering, Faculty of Chemical Technology, University of Chemistry and Technology, Technická 5, 166 28 Prague, Czech Republic

**Keywords:** magnesium potassium phosphate cement, heavy metals, immobilization, composite, structural and mechanical parameters, leaching, immobilization ratio

## Abstract

Heavy metal contamination, associated with the increase in industrial production and the development of the population in general, poses a significant risk in terms of the contamination of soil, water, and, consequently, industrial plants and human health. The presence of ecotoxic heavy metals (HMs) thus significantly limits the sustainable development of society and contributes to the deterioration of the quality of the environment as a whole. For this reason, the stabilization and immobilization of heavy metals is a very topical issue. This paper deals with the possibility of the simultaneous immobilization of heavy metals (Ba^2+^, Pb^2+^, and Zn^2+^) in mortar based on magnesium potassium phosphate cement (MKPC). The structural, mechanical, and hygric parameters of mortars artificially contaminated with heavy metals in the form of salt solutions were investigated together with the formed hydration products. In the leachates of the prepared samples, the content of HMs was measured and the immobilization ratio of each HM was determined. The immobilization rate of all the investigated HMs was >98.7%, which gave information about the effectiveness of the MKPC-based matrix for HM stabilization. Furthermore, the content of HMs in the leachates was below the prescribed limits for non-hazardous waste that can be safely treated without any environmental risks. Although the presence of heavy metals led to a reduction in the strength of the prepared mortar (46.5% and 57.3% in compressive and flexural strength, respectively), its mechanical resistance remained high enough for many construction applications. Moreover, the low values of the parameters characterizing the water transport (water absorption coefficient *A*_w_ = 4.26 × 10^−3^ kg·m^−2^·s^−1/2^ and sorptivity S = 4.0 × 10^−6^ m·s^−1/2^) clearly demonstrate the limited possibility of the leaching of heavy metals from the MKPC matrix structure.

## 1. Introduction

Heavy metals (HMs) pose a serious risk to soil, water, the atmospheric environment, human health, and crop yields [1,2], which has attracted many researchers to monitor and control HM pollution [3,4] and develop alternative safe treatment methods for HM-contaminated wastes and industrial by-products [5,6]. Due to the biological toxicity of HMs, i.e., bioaccumulation in living organisms and natural non-biodegradability [7], it is generally accepted that HM pollution negatively affects the sustainable development of society [8]. Presently, HM contamination is associated with the progressive and rapid industrialization and urbanization observed worldwide in recent decades, making it a global problem [9]. On the other hand, industrial pollution cannot be considered a modern phenomenon only. For example, ancient Rome and its imperial expansion two millennia ago brought an unprecedented intensification of mining and industrial activity, accompanied by the contamination of the air, land, and sea with toxic metals [10].

There are many sources of heavy metals in both terrestrial and aquatic environments. Besides natural HM sources, such as the weathering of natural geological sources and rocks [11,12] and air-borne dust in the atmosphere coming from forest fires, volcano eruptions, hydrothermal processes, and vegetation discharge [13,14], HMs have emerged as the result of anthropogenic activity, which is now considered a prevailing contaminant of the environment [8]. Fossil fuel combustion, mining, industrial production, transportation, agricultural activities, metallurgy, landfills, waste dumps, sewage sludge, and runoffs are the main “man-made” sources of HMs. As the mining industry supports most of the energy and industrial activities, it is considered to be one of the most harmful sources of waste contaminated with HMs [5]. In Europe, there are more than 500,000 sites contaminated by HMs coming from mining waste [15]. Xiao et al. reported that the situation is even worse, as the amount of polluted land is increasing annually by a rate of approx. 47,000 ha [16]. Wars, and in particular, the massive use of weapons, bombs, bullets, and even nuclear waste and residues, contribute to heavy metal pollution. A tragic and current example is what we are witnessing in Ukraine, which has always been the breadbasket of Europe and North Africa. Agriculture is also a significant source of HMs due to the use of pesticides, fertilizers, insecticides, etc. [10]. This can be at least partially mitigated by organic farming.

As the problems and serious risks associated with contamination by HMs have been extensively studied, several safe management methods for HMs have been developed and new, more effective, and environmentally friendly treatments are currently being studied. Immobilization methods such as cementation (cement solidification) [17,18,19] and thermal treatment techniques (sintering and vitrification) [20,21] are considered to be effective in converting HMs into more stable and less soluble phases that are no longer hazardous for the environment and humans.

However, the long-term environmental assessment of the risk of HM-contaminated solidified waste exposed to harsh environmental conditions is still unclear and needs to be studied on a case-by-case basis [22]. Also, the physical and chemical mechanisms of the immobilization of HMs using hydrated cements remain an open question, although they have been analyzed and characterized for many years [23,24]. As generally reported by Li et al. [25], the immobilization of HM ions in cementitious binders includes physical encapsulation, chemical incorporation, surface precipitation, and chemical substitution. Nevertheless, based on an analysis of the available literature, it is evident that further research is needed to clarify the solidification mechanisms and quantify the immobilization capacity [17].

Portland cement (PC), often enriched with various types of mineral additives, is the most widely studied binder for the immobilization of HMs. On the other hand, alkali-activated low-calcium aluminosilicate materials, known as geopolymers, and alkali-activated high-calcium materials are currently being studied extensively [26,27,28]. Similarly, different types of cements and their blends, such as magnesia-based binders, are the subject of ongoing research for the immobilization of HMs [29]. The reason for the study of PC alternatives for HM immobilization is the fact that some metal ions interfere with the hydration of PC, affecting the kinetics of ongoing reactions and the setting and hardening process, including a reduction in mechanical strength. This can lead to secondary contamination, which makes this disposal technology hazardous and tricky.

Magnesium potassium phosphate cement (MKPC) is one of the possible environmentally friendly substitutes for PC due to its lower carbon footprint and superior material properties, which are favorable for the treatment of HM-contaminated wastes, such as rapid strength development, a low porosity, and a high chemical stability. Compared to PC, MKPC possess a lower pH (typically 7–9), which can help to solidify metal ions and thus reduce the environmental burden of the solidified waste. To date, MKPC has not received much attention as a stabilization/immobilization matrix for the safe disposal of HMs compared to PC and geopolymers, despite its excellent densification, reduced leachability, higher propensity for binding with waste particles, and adaptability to environments with a wider range of pH values [30]. In a recent study, Cao et al. [31] investigated the lead–chlorine immobilization in composites composed of municipal solid waste incineration ash and MKPC. The research provided an in-depth understanding of Pb-Cl immobilization. The mechanisms of Zn and Cu immobilization in an innovative magnesium-sulfate-cementitious system were analyzed by Tan et al. [32]. The developed cementitious matrix showed a high immobilization capacity (~99%), i.e., low leaching toxicity. In addition, the solidification matrix yielded a high acid resistance. The identified immobilization principles were ionic substitution with Mg^2+^ and chemical complexation. In the review paper published by Tan et al. [33], magnesia-based cementitious materials were identified as a prospective solution for HM immobilization.

To contribute to the development of immobilization techniques based on alternative cements, the applicability of MKPC for the immobilization of HMs was evaluated. The immobilization ratio (immobilization efficiency) for specific HM ions was investigated in the present work, together with the effect of the HM contamination of MKPC-based mortar on its chemical structure, mineralogical composition, and basic physical parameters. The HMs studied were Ba^2+^, Pb^2+^, and Zn^2+^, and these were added to the MKPC mixture in the form of water-soluble salts. Pb^2+^ and Zn^2+^ are often found in the minerals of mining tailings. In our case, the soluble salts of heavy metals were used to artificially pollute the investigated composites.

## 2. Materials and Methods

The reference MKPC mortars were made of MgO powder (dead burnt, SMZ Jelšava, Jelšava, Slovakia); quartz sand (QS) of the 0–2 mm fraction (Filtrační písky, Chlum u Doks, Czech Republic); and chemicals of p.a. purity, KH_2_PO_4_ and Na_2_B_4_O_7_·10H_2_O (borax), which were produced by Lach-Ner, Neratovice, Czech Republic. Based on our previous research on MKPC-based composites [34], borax was used as a setting retarder to ensure the workability of the fresh mix and its casting into molds. The heavy metals were artificially mixed into the MKPC mortar mixture in the form of soluble salts. Barium chloride dehydrate (BaCl_2_·2H_2_O), lead nitrate (Pb(NO_3_)_2_), and zinc chloride (ZnCl_2_) were produced by Lach-Ner, Neratovice, Czech Republic. The dosage of soluble salts was 2.5 wt.% of the binder. Tap water was used to mix the samples. The composition of the prepared mortars is introduced in Table 1. The reference (control) mortar was labeled MKPC-Ref and the HM-contaminated mortar was labeled MKPC-HM. Except for the addition of HMs, the composition of both mortars was similar, i.e., the w/b ratio was 0.25, the MgO/KH_2_PO_4_ molar ratio was 8, and the mass ratio of sand to binder was 1.

A planetary-type mixer (ELE International, Milton Keynes, UK) was used for the preparation of the samples. MgO, KH_2_PO_4_, borax, and sand were mixed first for 1 min using a low-speed regime. The batch water and salts were then added and the formed blend was stirred for another 1 min (paddle: 140 rpm and mixing head: 62 rpm). The mixing was then stopped and the mixer head and boll were cleaned, followed by hand-mixing for 1 min. Finally, the mixture was homogenized for 1 min at a high speed (paddle: 285 rpm, mixing head: 125 rpm). Each salt was dissolved in 1/3 of batch water separately. ZnCl_2_ was well soluble in water. On the other hand, the Pb(NO_3_)_2_ and BaCl_2_·2H_2_O solutions were heated at 60 °C to uniformly dilute the salts.

The fresh mixtures were cast into prismatic molds (40 × 40 × 160 mm). After 24 h, these were demolded and freely cured in the laboratory for 27 days. The laboratory temperature and relative humidity were kept constant at 23 ± 2 °C and 40 ± 5 RH%.

**Figure 1:**
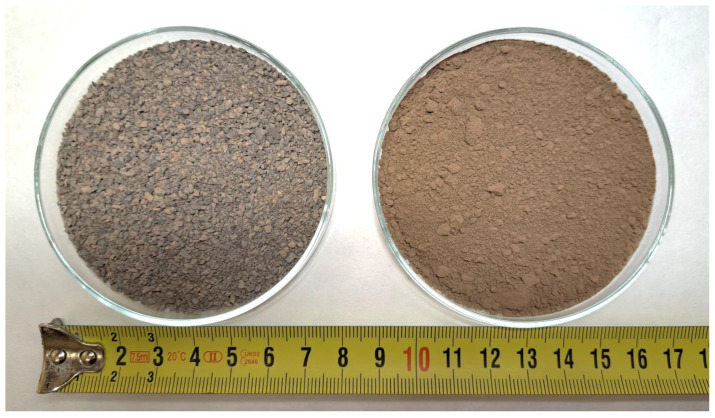
Dead burnt magnesia: as commercially delivered (**left**) and grinded by a ball mill (**right**).

A ball mill was used for grinding the originally delivered dead burnt magnesia. The grinding of the original MgO supplied by the manufacturer was carried out until the resulting magnesium dust was no longer refined, to ensure the reactivity of the magnesium binder. This was verified by measuring the particle size distribution with laser diffraction. The original MgO and the powdered form are shown in Figure 1.

**Figure 2:**
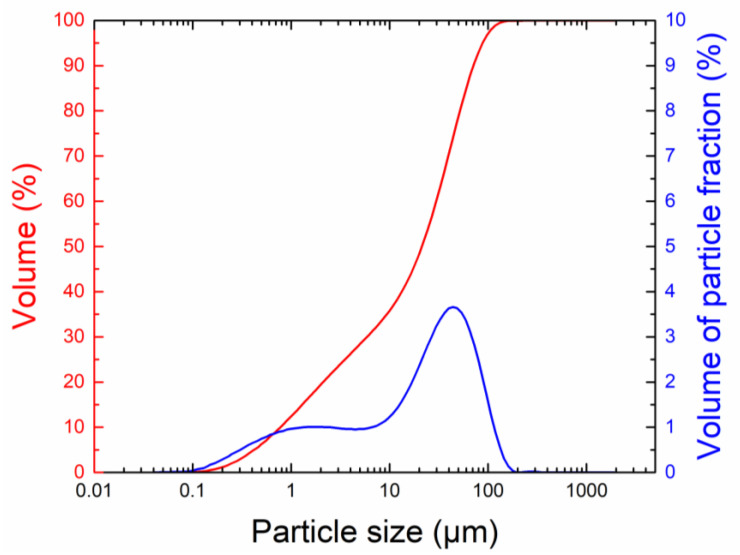
Particle size of milled magnesia powder.

The particle size distribution of the powdered magnesia, measured with a laser diffractometer (Analysette 22 Micro-Tech plus, Fritsch, Idar-Oberstein, Germany), is shown in Figure 2. The maximum particle size was 177 μm, d_90_: 72.4 μm, and d_50_: 20.1 μm. An ED-XRF spectrometer (ARL QUANT’X, Thermo Fischer Scientific, Waltham, MA, USA) was used to determine the chemical composition of the dead burnt magnesia. The X-ray voltage power generated up to 50 kV and up to 2 mA with a 50 W power limitation. A rhodium (Rh) X-ray tube target with a Be window and a vacuum environment was used for the analysis. The main substances identified in the magnesia powder are presented in Table 2.

**Figure 3:**
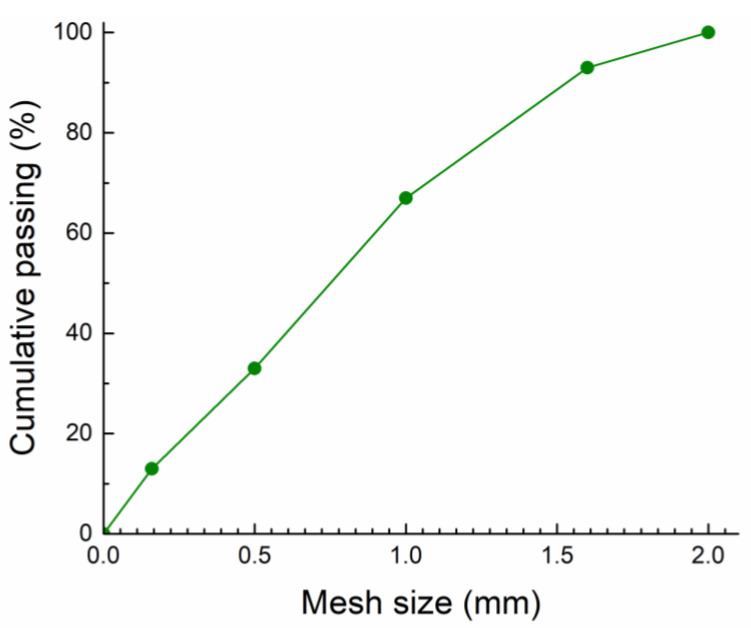
Particle size curve of quartz sand.

The fineness modulus of quartz sand was 2.2. Its particle size curve is graphed in Figure 3.

The physical properties measured for the hardened MKPC samples, the experimental methods used, and the expanded combined uncertainties (ECUs) of the assessment of each parameter are summarized in Table 3. The microstructure of the researched materials was analyzed using mercury intrusion porosimetry (MIP). The samples were vacuum-dried and placed in a Pascal series porosimeter set (140 + 440, Thermo Scientific, Waltham, MA, USA). The sample mass was approximately 1.5 g.

To determine the immobilization ratio of HMs, a leaching test was carried out. It was performed according to EN 12457-2 [38]. In the leaching test, the liquid/solid ratio was 10 L/kg. Both distilled water and rainwater were used as solvents. The leachates were stirred at a frequency of 175 rpm for 24 h and the concentration of heavy metals was measured using atomic adsorption spectrometry (AAS) with an ICE 3000 Series AA spectrometer (Thermo Scientific). Except for the leaching of HMs, the stability of the MKPC phase was characterized by measuring the concentration of Mg and K in the prepared leachates. The pH and electrical conductivity of the leachate were also investigated. The pH was measured with a pH/ION 340 (WTW, Xylem Analytics, Weilheim, Germany) equipped with a SenTix 21 (WTW) pH electrode. The electrical conductivity of the leachate was measured using a pH/Cond 3320 m (WTW) and a TetraCon 325 sensor (WTW, Xylem Analytics, Weilheim, Germany).

The phase composition was studied using XRD (CuKα, λ = 0.15418 nm) with a Bruker D2 Phaser (Bruker AXS GmbH, Karlsruhe, Germany) with Bragg–Brentano geometry. The XRD measurements utilized an applied voltage of 30 kV and a current of 10 mA. The step size for data collection was set to 0.02025° (2θ), and the angular range covered was 5° to 80°. The morphology was analyzed using SEM via a Tescan Lyra (Tescan, Brno, Czech Republic) dual-beam microscope equipped with a field-emission gun (FEG) electron source. An elemental composition analysis and mapping were performed using an energy-dispersive spectroscopy (EDS) analyzer, specifically the X-MaxN model, featuring a 20 mm^2^ silicon drift detector (SDD) manufactured by Oxford Instruments (Oxford, UK). The thermal stability was analyzed using STA-MS with a thermal analyzer (Setaram Setsys Evolution, Caluire, France; operating range of 25–1600 °C) and an OmniStarTM quadrupole-type mass spectrometer (Pffeifer Vacuum, Aßlar, Germany) in a dynamic air atmosphere using synthetic air (without CO_2_) at 50 mL/min and with a heating rate of 10 °C/min.

In addition, for selected analyses, pastes without quartz sand were also prepared. These samples were used mainly for the analysis of the phase composition, the identification of hydrated products, and the analysis of the thermal performance, as the presence of silica sand could confuse the interpretation of the obtained data. These samples were termed MKCP-REF-PASTE and MKCP-HM-PASTE.

FTIR spectroscopy was used to detect the hydration products in the MKPC-HM-PASTE samples with incorporated heavy metals in comparison to the MKPC-REF-PASTE control samples. Dry samples were first crushed and then homogenized to a powder using a MM 400 ball mill (RETSCH, Haan, Germany). The infrared spectra were obtained using a Nicolet 6700 spectrometer (Thermo Fisher Scientific, Waltham, MA, USA) with an attenuated total reflectance (ATR) sampling tool. The samples were scanned in the mid-infrared (MIR) region from 4000 to 400 cm^−1^ with a spectral resolution of 4 cm^−1^ on a diamond crystal (32 scans). The deconvolution of the bands, fitted with a Gaussian function, in the IR spectra was performed using Thermo Fisher Scientific software OMNIC 8.3.103, 1992–2011.

## 3. Results and Discussion

**Figure 4:**
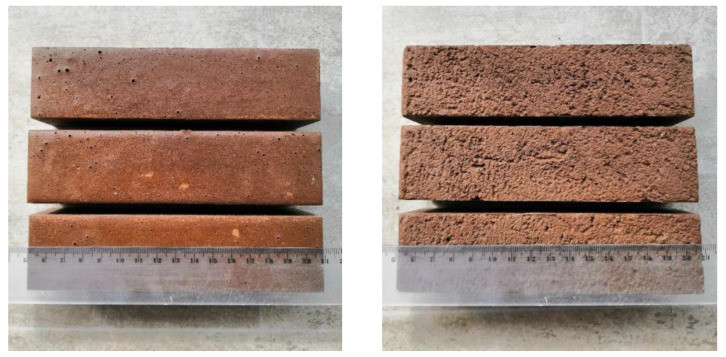
The 28-day samples (40/40/160 mm prisms) prepared for testing (**left**—MKPC-REF; **right**—MKPC-HM).

The 28-day samples of MKPC prepared for testing are shown in Figure 4. In this paper, for these samples, a complete chemical and physical characterization was carried out to demonstrate the applicability of the MKPC matrix for the stabilization of HMs and to access the effect of the present HMs on the properties of the composites under investigation.

**Figure 5:**
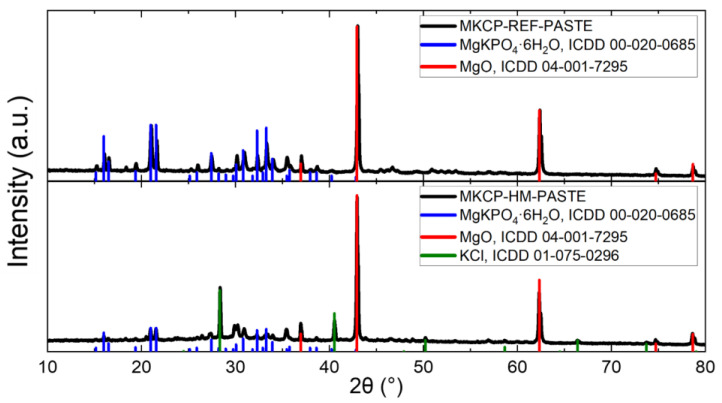
XRD patterns of MKCP-REF-PASTE and MKCP-HM-PASTE.

The MKPC samples were analyzed using XRD; unfortunately, because of the very-high-intensity reflections of SiO_2_, almost none of the other phases were reliably detectable. This issue was overcome by preparing and analyzing cement paste samples without SiO_2_. In the MKCP-REF-PASTE sample, two phases were observed—MgKPO_4_∙6H_2_O (ICDD 00-020-0685) and unreacted MgO (ICDD 04-001-7295), as it was used in excess over the stoichiometry (see Figure 5). In the MKCP-HM-PASTE sample, three phases were detected—the same phases as in the reference sample and KCl (ICDD 01-075-0296), which was caused by the addition of HMs in the form of soluble chlorides. Newly formed phases with HMs (PbHPO_4_, Ba_5_(OH)(PO_4_)_3_, and Zn(OH)_2_) were not detected via XRD, most probably because of the low concentrations of HMs or the low crystallinity of the HM phases.

**Figure 6:**
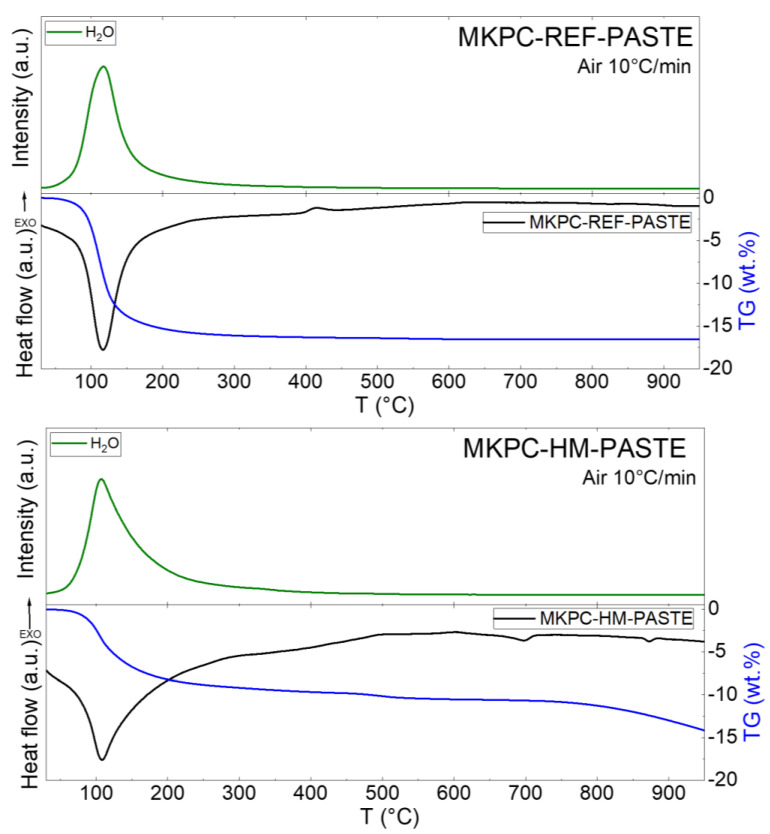
STA-MS for MKCP-REF-PASTE and MKCP-HM-PASTE.

In the next step, STA-MS was used to confirm the presence of the magnesium phosphate phase in the pastes. The DTA, TG, and MS curves for released water are shown in Figure 6. For both samples, a major endothermic effect was found in the temperature range between 100 and 150 °C. This effect was caused by the water release from the MgKPO_4_∙6H_2_O phase (K-struvite), which is also clearly visible from the MS spectrum. In the MKCP-HM-PASTE sample, there were two visible endothermic effects at ~700 °C and ~875 °C accompanied by a mass loss that could be assigned to the decomposition of the HM phases. Unfortunately, this theory was not confirmed by the literature because of low amounts of the HM phases.

**Figure 7:**
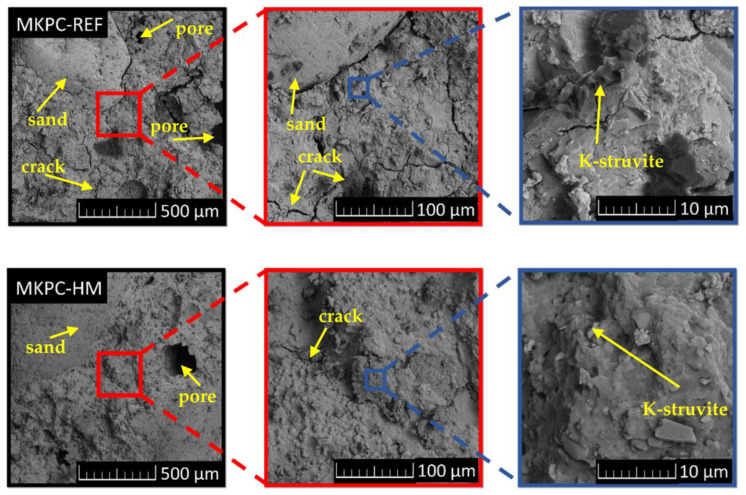
SEM micrographs of MKCP-REF and MKCP-HM at various magnifications.

The SEM measurement of MKPC-based mortars showed quite a uniform surface with cracks of various dimensions, pores, and irregular sand particles. Those cracks could be partly a result of breaking a large sample to obtain a small piece that would fit into the microscope and volume changes in the MKPC matrix during setting and hardening. Nevertheless, the microstructure of both composites can be considered dense, thus contributing to the immobilization of HMs. The highest-magnification SEM images showed traces of columnar crystals of K-struvite. All the micrographs at various magnifications are depictured in Figure 7.

**Figure 8:**
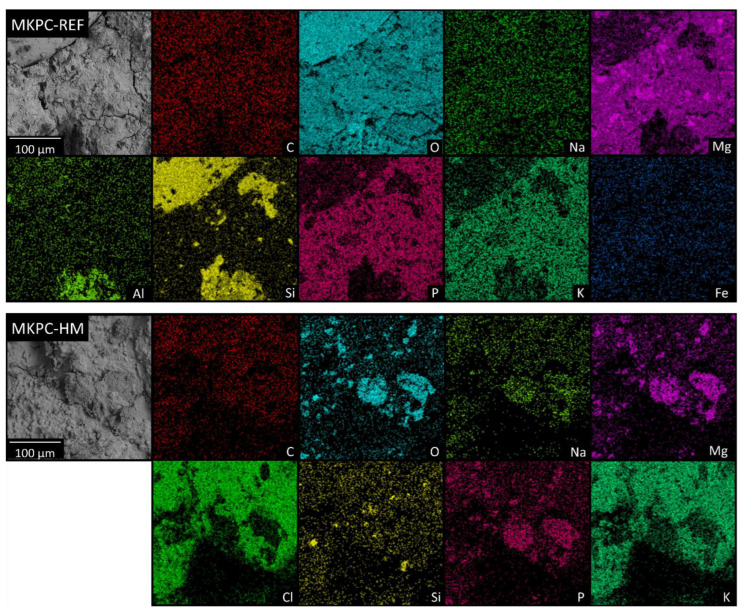
EDS elemental maps of MKPC-REF and MKPC-HM.

Moreover, the elemental composition of MKPC-based mortars was analyzed using EDS. The elements of the MKCP phase—Mg, K, P, and O—were clearly detected across the whole surface. Si and Mg were detected in cluster-like shapes because of the much larger dimensions of the particles of SiO_2_ and the unreacted MgO. Also, C, Al, Na, Ca, and Fe were detected as impurities of the starting materials, but in much lower concentrations (see Table 4). The sample MKPC-HM contained K and Cl from the KCl, as determined using XRD. The obtained elemental maps of both samples can be seen in Figure 8. Such a high content of KCl on the surface can be explained by the subsequent crystallization of KCl crystals from the solution in pores and other defects. Let us note that the EDS mapping was performed on the fracture surface; therefore, the detected content of KCl was higher for EDS than for XRD, where the powder sample was analyzed. As the KCl was on the top of the analyzed surface, elements with a lower signal such as Al, Fe, and Ca were not detected in the MKCP-HM sample, even though they were surely present.

**Figure 9:**
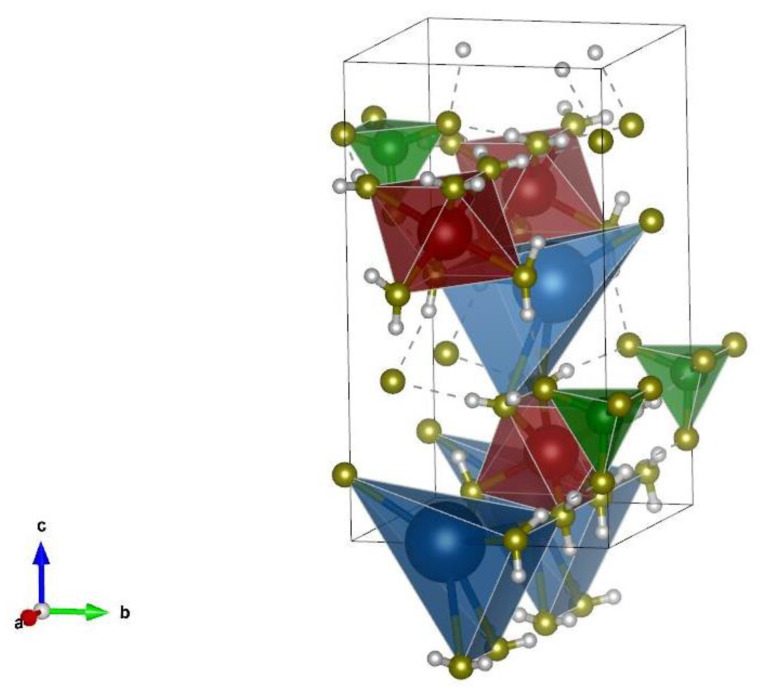
Structure of K-struvite: K—blue, Mg—red, P—green, O—yellow, and H—grey.

**Figure 10:**
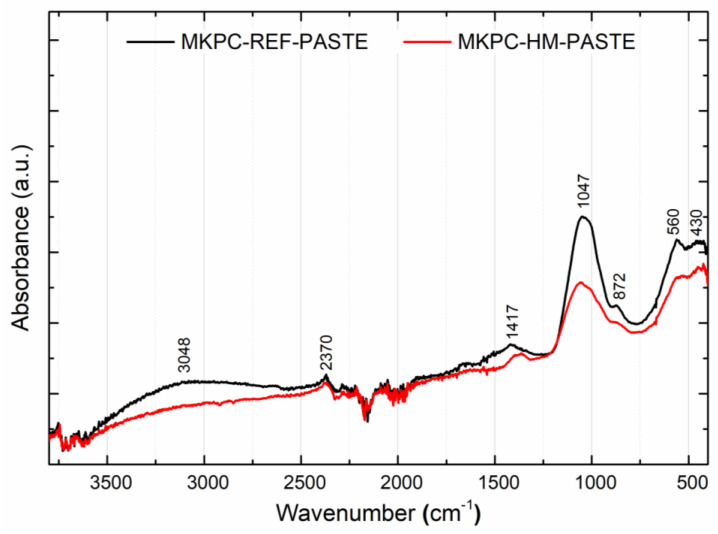
IR spectra of MKPC-REF-PASTE and MKPC-HM-PASTE.

The mixed proportion of MKPC had a great influence on the composition of the hydration products, especially in the case of heavy metal incorporation into the matrix. The presence of magnesium potassium phosphate hexahydrate (MgKPO_4_∙6H_2_O, MKP) is considered to be the bearer of strength [39]. The structure of K-struvite is shown in Figure 9. To obtain information on the hydration products, FTIR spectroscopy was applied. The obtained infrared spectra are presented in Figure 10. A summarization of the major absorption band assignments is given in Table 5.

The analyzed MKPC-PASTE sample spectra certainly included the characteristic absorption bands coming from the fundamental vibrations of phosphate cement and structural water. Stretching vibrations above 3600 cm^−1^ are typical for O-H bonds in brucite coming from the following reaction:

$$Mg^{2+}+2H_{2}O\to Mg{\left(OH\right)}_{2}+2H^{+}.$$

In our case, the characteristic absorption band was visible at 3753 cm^−1^ [40]. A comparison of the intensities, which are a measure of the amount of brucite, showed that the formation of brucite during hydration in the MKPC-HM pastes was three times higher than in the reference paste. The broad absorption band between 3500 and 3000 cm^−1^, with the maximum at 3048 cm^−1^, was attributed to the stretching vibration of O-H bonds in the water hydroxyl group. The band intensities at 2370 cm^−1^ were the stretching vibrations of H-O in the K-struvite crystalline water [41]. The characteristic absorption bands of the C=O stretching vibration in carbonates at 1417 cm^−1^ were due to the presence of MgCO_3_ from the carbonation reaction [42]:

$$Mg{\left(OH\right)}_{2}+CO_{2}\to MgCO_{3}.$$

K-struvite was formed during MKPC hydration according to the following reaction:

$$K^{+}+Mg^{2+}+HPO_{4}^{2-}+6H_{2}O\to KMgPO_{4}\cdot 6H_{2}O.$$

The characteristic vibration originating from phosphate units due to the presence of K-struvite was observed at 1047 cm^−1^ [41]. A comparison of the intensities showed a lower amount of K-struvite in the case of MKPC-HM-PASTE, which corresponded to the measured mechanical properties. Finally, stretching vibrations of Mg-O bonds were found at 540 and 430 cm^−1^ [40,43].

To identify the possible bond vibrations coming from heavy metal compounds created within the hydration of the MKPC paste, the pure components were synthetized according to the following reactions:

$$Pb^{2+}+HPO_{4}^{2-}+H_{2}O\to PbHPO_{4}+OH^{-},$$

$$5Ba^{2+}+{\left(HPO_{4}^{2-}\right)}_{3}+H_{2}O\to Ba_{5}\left(OH\right){\left(PO_{4}\right)}_{3}+H^{+},$$

$$Zn^{2+}+2H_{2}O\to Zn{\left(OH\right)}_{2}+2H^{+},$$

and characteristic bond vibrations were searched [20]. The $M^{2+}$-O bond stretching vibration was detected at around 1610 cm^−1^, which confirmed the formation of metal phosphates during hydration. Other characteristic vibrations agreed with or overlapped on the bond vibrations identified for the MKPC pastes. ${\mathrm{Pb}}^{2+}$ and ${\mathrm{Ba}}^{2+}$ formed solid phases (lead (II) hydrogen phosphate and barium hydroxylapatite), while ${\mathrm{Zn}}^{2+}$ remained present as part of the pore solution.

**Figure 11:**
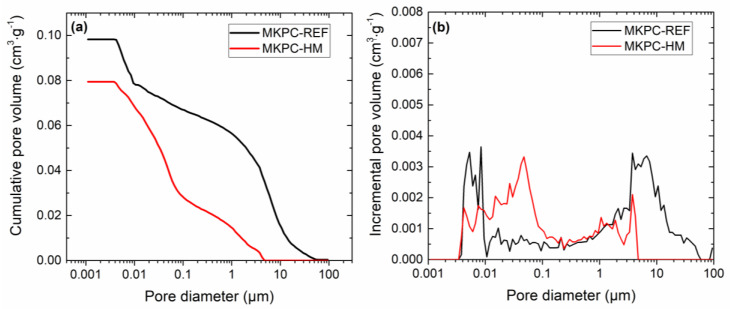
Pore size distribution: (**a**) cumulative pore volume and (**b**) incremental pore volume.

The macro-structural and micro-structural parameters of the examined MKPC mortars are introduced in Table 6. The bulk density remained almost unchanged by the incorporation of heavy metal salts in the composition of the composite. The drop in specific density was approx. 3.0% compared to the control mortar, MKPC-R. The changes in both densities resulted in a decrease in the porosity of the composite with HMs, which was ~8.8%. The data of the macro-structural parameters corresponded well to the results provided by MIP, i.e., the total pore volume, the average pore diameter, and the cumulative pore volume curves (Figure 11).

**Figure 12:**
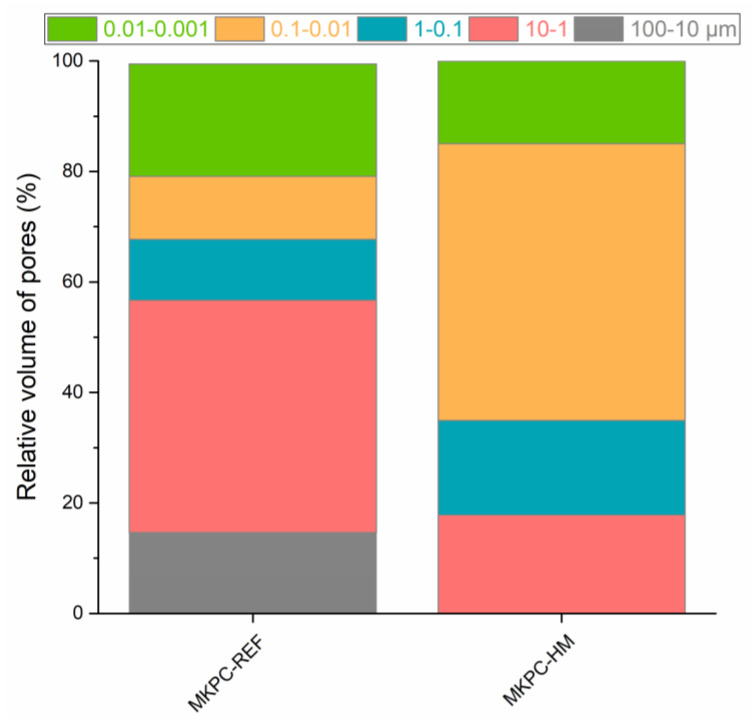
The proportion of pores identified in the studied MKPC mortars.

The solidification of the MKPC matrix contaminated with HMs is well illustrated in Figure 11b (incremental pore size distribution) and Figure 12, where the relative pore volume of a specific pore diameter range is shown. Compared to the control mortar, MKPC-HM was characterized by a lower volume of pores in the 10–100 μm, 1–10 μm, and 0.001–0.01 μm diameter ranges. In contrast, the proportion of pores with diameters of 0.1–1 μm and 0.01–0.1 μm was higher. With regard to the limitation of heavy metal leaching in a physical way, i.e., encapsulation, a reduction in the volume of capillary pores enabling liquid water transport is essential. Capillary pores with diameters ranging from 0.1 μm to 10 μm are considered to be responsible for bulk water transport [44]. The total volume of these pores was 53.1% for MKPC-R and 34.9% for MKPC-HM. The decrease of approximately 34% in the volume of active capillary pores apparently documented the retardation of water transport in the MKPC-HM matrix, thus contributing to the immobilization of HMs.

The determined parameters characterizing the moisture transport in the prepared composites are shown in Table 7. In agreement with the pore size distribution data, i.e., the decrease in the volume of capillary pores for mortars contaminated with HMs, the water absorption coefficient and sorptivity were significantly reduced for MKPC-HM compared to the reference material, MKPC-R. The water absorption *A_w_* decreased by 57.3% and the sorptivity S was reduced by approximately 60%, which makes a good assumption about the decrease in the HM leachability. The reduction in the water transport ability due to the refinement of the porous structure of the MPC-HM samples can be attributed to the newly formed HM compounds precipitated during MKPC hydration and HM-induced changes in the crystalline phase of MKPC. The compounds forming the hardened structure of the examined composites were analyzed in detail and identified using FTIR spectroscopy (see above).

The pH and electrical conductivity data of the leachates are presented in Table 8. For comparison, the data measured for distilled water and rainwater are also presented. The decreased pH of the distilled water was the result of the effect of CO_2_ from the atmosphere. The distilled water showed normal values. The pH values of the leachates from the reference samples and those containing HMs met the limits prescribed by Czech Regulation No. 8/2021 (catalogue of wastes and assessment of their properties) [45], and in this respect, they did not show the hazardous property H15, i.e., the ability to release dangerous substances into the environment. The electrical conductivity values of the leachates prepared from the mortars containing heavy metals increased significantly compared to the values obtained for the reference mortars. This was due to the presence of added salts. The limit value for electrical conductivity prescribed by Czech Regulation No. 70/2018 is 1250 μS/cm, which is much lower than the values obtained for the reference mortar. It is expected that the high pH and electrical conductivity were affected not only by the presence of heavy metal salts, but also by some limited water-induced damage to the MKPC matrix. Therefore, the concentration of K and Mg ions in the leachates was additionally measured using AAS. The data are presented in Table 9 and show an increased dissolution of the formed MKPC matrix when the samples were leached in rainwater compared to their leaching in distilled water. In addition, the leaching of K and Mg was further enhanced by the contamination with HMs. The high concentration of leached K can be attributed to the dissolution of the KCl formed during the precipitation of the MKPC matrix in the presence of artificially added HMs. However, since K and Mg ions are not considered hazardous to the environment, their concentration in the leachates cannot be considered quantitatively significant.

The concentration of HMs in the particular leachates, the corresponding safe limit value, and the achieved immobilization ratio are summarized in Table 10, Table 11 and Table 12. Safe concentrations of heavy metals in the leachate are marked in green, and concentrations exceeding the prescribed limit according to Regulation No. 8/2021 [45] are highlighted in red. The MKPC matrix showed its high efficiency in the simultaneous immobilization of HMs, namely Pb, Zn, and Ba ions. In the case of leaching in distilled water, the immobilization ratio was >99.7%, which is really high, and the concentration of the respective metals in the leachates was safely below the prescribed limits. Leaching in rainwater greatly increased the concentration of HMs in the leachates, i.e., the immobilization ratio was lowered, but still exceeded 98.7%. The concentration of Pb was higher than the limit value [45], but one must consider that an extremely high content of HMs was added to the MKPC mixture, which is unlikely to happen in the real environment. The high immobilization ratio was the result of the low solubility of the heavy metal phosphates that are preferentially formed during their reaction with the MKPC binder [46,47], which well demonstrated the FTIR results presented above.

**Figure 13:**
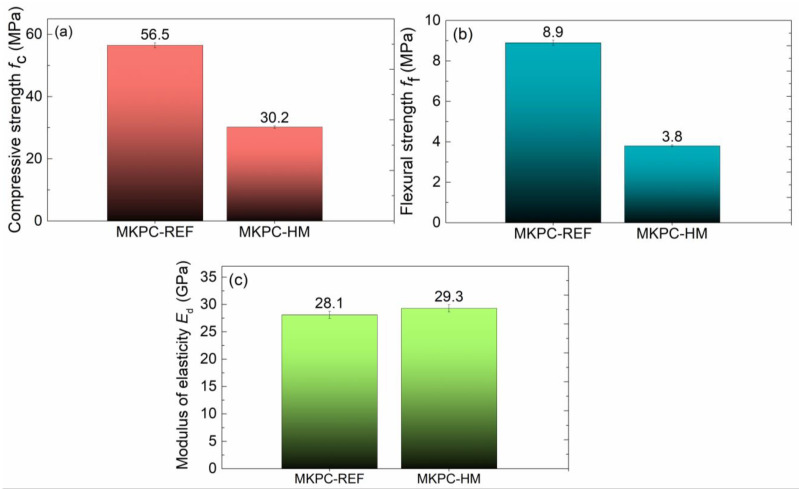
Mechanical parameters of the studied MKPC mortars: (**a**) compressive strength, (**b**) flexural strength, (**c**) modulus of elasticity.

The results of the mechanical parameter tests are shown in Figure 13.

The stiffness of both MKPC mortars, characterized by Young’s modulus, was similar, typically in the range of the expanded combined uncertainty of the dynamic Young’s modulus assessment. On the other hand, the presence of HMs was highly detrimental to the mechanical strength. The decrease in compressive strength was approximately 46.5% and that in flexural strength was 57.3% compared to the control MKPC-R sample. Since the material MHPC-HM exhibited a more condensed structure of reduced porosity and without the presence of macropores >10 μm, the decrease in mechanical strength cannot be attributed to the physical effect of the presence of HMs, but it evidences the chemical nature of the structural changes in MKPC. A significant drop in the strength of MKPC was observed, e.g., by Buj et al. [48] and Wang et al. [49]. The authors stated that the decrease in compressive strength increased with the concentration of HMs in the MKPC matrix, which is detrimental to its S/S (stabilization/solidification) ability. The decrease in the mechanical strength was caused by the formation of heavy metal phosphates, i.e., changes in the reaction of the MKPC binder, that reduced the amount of K-struvite (MgKPO_4_·6H_2_O), which is the bearer of the MKPC strength [50,51]. This was well visible in the changes in the intensity peak of magnesium potassium phosphate hexahydrate (K-struvite) presented in the IR spectra (Figure 9). Except for the lowering of the K-struvite content, the presence of HMs can lead to defects in the crystalline phase of the MKPC matrix, for example, through the encapsulation of the formed HM hydrogen phosphate or apatite. However, the HMs were not the only cause of the strength degradation. The formation of KCl was another significant effect that weakened the mechanical properties of the HKPC-HM mortars. Not least of which, the formation of substantial amounts of brucite (MgO) in HKPC-HM, which was well documented by the FTIR analysis, led to the worsening of mechanical resistance [52]. This can be partially compensated for by continuous carbonation, as the formation of carbonates (magnesite, nesquehonite, hydromagnesite, etc.) may densify the microstructure of the interfacial transition zone between the aggregate and the MKPC matrix [53,54]. Although the deterioration in mechanical resistance was significant, the retained compressive strength remained high enough for different prospective structural applications of the MKPC-HM composite.

## 4. Conclusions

The effect of the presence of artificially added HMs in MKPC mortar on its properties was experimentally investigated, together with the ability of an MKPC matrix to immobilize Ba^2+^, Pb^2+^, and Zn^2+^ ions simultaneously. Based on the obtained results, it can be concluded that an MKPC matrix represents an effective alternative for the stabilization/immobilization of HMs, from both its physical and chemical principles. The physical effect of MKPC lies in its low ability to transport water, thus limiting the leachability of HMs. The chemical effect of MKPC in the stabilization of HMs results from the formation of insoluble compounds that are formed during the reaction of MKPC components and HMs. Since the application potential of MKPC in the stabilization/immobilization of HM ions was verified and experimentally proven, the MKPC binder will be exploited in future work for the possible immobilization of HMs present in industrial by-products and wastes. In follow-up research, specific attention will be paid not only to the immobilization ratio of HMs, but also to the low carbon footprint of the final composites and their engineering properties. In this research, the long-term immobilization effect will be examined with respect to composite durability.

## Figures and Tables

**Table 1:** The composition of the studied MKPC mixtures (g).

Sample	MgO	KH_2_PO_4_	Water	QS	Borax	ZnCl_2_	BaCl_2_·2H_2_O	Pb(NO_3_)_2_
MKPC-R	843.1	272	229.3	3 × 305.7	45.9	-	-	-
MKPC-HM	843.1	272	229.3	3 × 305.7	45.9	47.8	40.8	36.7

**Table 2:** The chemical composition of magnesia expressed in the form of oxides.

MgO	Fe_2_O_3_	Al_2_O_3_	CaO	SiO_2_	MnO	SO_3_	K_2_O
76.50	12.55	5.76	3.32	1.04	0.69	0.11	0.03

**Table 3:** Summary of investigated physical parameters, used methods, and ECUs.

Property, Parameter	Symbol	Unit	ECU (%)	Standard/Method
Bulk density	*ρ*_b_	kg·m^−3^	1.4	gravimetry
Specific density	*ρ*_s_	kg·m^−3^	1.2	helium pycnometry
Total open porosity	*ψ*	%	2.0	pycnometry/gravimetry
Flexural strength	*f*_f_	MPa	1.4	EN 1015-11 [35]
Compressive strength	*f*_c_	MPa	1.4	EN 1015-11 [35]
Young’s modulus	*E*_d_	GPa	2.3	ultrasonic velocity
Water absorption coefficient	*A*_w_	kg·m^−2^·s^−1/2^	1.2	1-D free water uptake test [36,37]
Water sorptivity	*S*	m^2^·s^−1/2^	1.2	1-D free water uptake test [36,37]

**Table 4:** Elemental composition of MKCP-REF and MKCP-HM via EDS in wt.%.

Sample	C	O	Na	Mg	Al	Si	P	K	Fe	Cl	Ca
MKPC-REF	5.4	43.2	0.7	15.0	1.1	12.2	6.8	12.2	2.3	-	1.2
MKPC-HM	8.6	15.5	0.5	3.2	-	0.9	1.0	38.6	-	31.7	-

**Table 5:** Assignments of the major absorption bands of MKCP-REF-PASTE and MKCP-HM-PASTE.

Wavenumber/cm^−1^	Assignment
3048	ν (-O-H) in water
2370	ν (-O-H) of water molecules clustered in crystalline form in K-struvite (MgKPO_4_·6H_2_O)
1417	ν (-C=O) in magnesite (MgCO_3_)
1047	ν (-P=O) in phosphates (PO_4_^3−^)
872	ν (K-O-P-, Mg-O-P-) in K-struvite (MgKPO_4_·6H_2_O)
560, 430	ν (Mg=O, Mg-O-H) in periclase (MgO) and brucite (Mg(OH)_2_)

**Table 6:** Structural parameters of MKPC samples.

Mortar	Bulk Density ρ_b_ (kg·m^−3^)	Specific Density ρ_s_ (kg·m^−3^)	Total Open Porosity *φ* (%)	Total Pore Volume (cm^3^·g^−1^)	Average Pore Diameter (μm)
MPC-REF	2247 ± 31	2867 ± 34	21.6 ± 0.4	0.0988	0.0249
MPC-HM	2231 ± 31	2780 ± 33	19.7 ± 0.4	0.0795	0.0224

**Table 7:** Hygric parameters of MKPC samples.

Mortar	Water Absorption Coefficient *A_w_* ×10^−3^ (kg·m^−2^·s^−1/2^)	Sorptivity *S* ×10^−6^ (m·s^−1/2^)
MPC-REF	9.98 ± 0.12	10.0 ± 0.1
MPC-HM	4.26 ± 0.05	4.0 ± 0.1

**Table 8:** The pH and electrical conductivity of the leachates and used solvents.

Solvent, Leachate	pH (-)	Limit Value of pH [39] (-)	Electrical Conductivity (μS·cm^−1^)
Distilled water (D)	7.00	-	6.0
Rainwater (R)	6.41	-	31.2
MKPC-R-D	11.18	5.5 < 11.18 < 13.00	3800.0
MKPC-R-R	11.14	5.5 < 11.14 < 13.00	3780.0
MKPC-HM-D	10.07	5.5 < 10.07 < 13.00	8280.0
MKPC-HM-R	10.07	5.5 < 10.07 < 13.00	8280.0

**Table 9:** Concentration of Mg and K in the leachates and in the used solvents.

Solvent, Leachate	Concentration of K (mg·L^−1^)	Concentration of Mg (mg·L^−1^)
Distilled water (D)	n.d.	0.0013
Rainwater (R)	0.8812	0.5103
MKPC-R-D	804.4099	13.3010
MKPC-R-R	886.5471	63.0548
MKPC-HM-D	3979.1110	116.6638
MKPC-HM-R	2161.0330	184.2604

**Table 10:** Concentration of Pb in the leachates.

Sample	Concentration of Pb^2+^ (mg·L^−1^)	Concentration Limit [39] (mg·L^−1^)	Immobilization Ratio (%)
MKPC-R-D	0.0001	5	n.d.
MKPC-R-R	0.0435	5	n.d.
MKPC-HM-D	1.3134	5	99.86
MKPC-HM-R	11.3471	5	98.76

**Table 11:** Concentration of Zn in the leachates.

Sample	Concentration of Zn^2+^ (mg·L^−1^)	Concentration Limit [39] (mg·L^−1^)	Immobilization Ratio (%)
MKPC-R-D	0.0749	20	n.d.
MKPC-R-R	0.0163	20	n.d.
MKPC-HM-D	1.2323	20	99.87
MKPC-HM-R	10.3513	20	98.87

**Table 12:** Concentration of Ba in the leachates.

Sample	Concentration of Ba^2+^ (mg·L^−1^)	Concentration Limit [39] (mg·L^−1^)	Immobilization Ratio (%)
MKPC-R-D	0.0005	30	n.d.
MKPC-R-R	0.0365	30	n.d.
MKPC-HM-D	2.4331	30	99.74
MKPC-HM-R	4.2469	30	99.54

## Data Availability

The data presented in this study are available from the corresponding author upon request.
